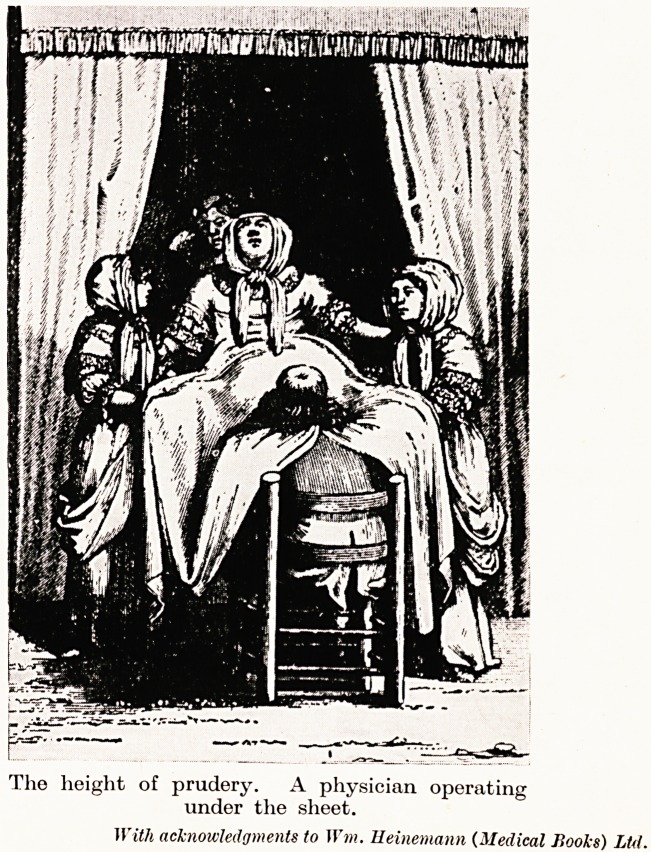# Obstetrics and Gynæcology Old and New

**Published:** 1949-01

**Authors:** H. J. Drew Smythe

**Affiliations:** Professor of Obstetrics in the University of Bristol


### The Bristol Medico-Chirurgical Journal

*A Journal of the Medical Sciences for the*

### West of England and South Wales

*" Scire est nescire, nisi id me*
*Scire alius sciret."*

JANUARY, 1949

### OBSTETRICS AND GYNÆCOLOGY OLD AND NEW

The Presidential Address, delivered on Wednesday, 13th October, 1948, at the opening of the Seventieth Session of the Bristol Medico-Chirurgical Society

BY

H. J. DREW SMYTHE, M.C., T.D.

*Professor of Obstetrics in the University of Bristol*

IN this address I want to take you from ancient to modern obstetrics and gynæcology, not to compare them but rather as medical history. It is said that the primitive races had little difficulty in childbirth, and that the same applies to uncivilized races in these days. This I doubt very much, having seen deliveries and the result of such deliveries in uncivilized races in the East. From ancient books of midwifery one reads of the means by which nature was encouraged to do her best, or worst. Any difficulty in the delivery of a primitive woman was not considered to be one of obstetrics, but due to the unwillingness of the child to be born. The child, therefore, for its unwillingness had to be destroyed, and needless to say the mother usually shared the same fate. Special instruments for destroying the child *in utero* were designed for this purpose.

The posture assumed by the primitive woman for the birth of the child was the squatting position, which is the natural position for bearing down. This position, or variations, have been used throughout the ages up to the present day. The first variation of this position was for the patient to sit between the knees of one woman who clasped the patient round the waist, while another woman held the patient's bended knees. The next step was the introduction of the obstetric chair, which was part of the professional equipment of every midwife. This chair is mentioned in the first chapter of Exodus, when Pharaoh commands the midwives to slay all Jewish male infants : "When you do the office of midwife to the Hebrew women and see them upon the stool." The chair remained the necessary equipment of a midwife up to the nineteenth century. In the seventeenth century, however, Mariceau in France introduced the delivery of the patient in bed, though the midwives still used the obstetric chair with its attendant source of sepsis.

In more modern times a variation of this chair was the use of a commode during the first and early second stage of labour, which I hope has now been abandoned. The squatting position is still used in modern midwifery, but the patient lies on her back in bed with the knees drawn up. A different variation is used at the present time during the second stage of labour. The position is the same, but the patient, lying on her back or on her side, clasps the knees with the hands until the actual delivery of the head.

Many changes in the technique of delivery have taken place during the ages. It is true that primitive women were in a better position to have a normal labour than a large number of women in modern times, as they did not undergo the risks of civilization, namely, overcrowding, incorrect feeding in childhood, and sepsis at delivery. In spite of this, obstetric difficulties were bound to arise, and in ancient days assistance was of a direct nature. The patient was held up by the feet and well shaken, or rolled or bounced in a blanket. Other methods were to trample on the patient's abdomen : or the patient was hung up by the arms to a tree, while those assisting her bore down on a strap placed over the fundus of the uterus. Amongst the Red Indians the patient was placed out in the open plain, and a warrior galloped straight at her—avoiding the patient, we hope, at the last minute. This presumably was for its psychological effect. Then we have the medicine-man of a tribe, who would mumble some incantation, anoint the woman with saliva, or use some gruesome relic to do so : but he did not use any aids to midwifery, and the outcome was more or less successful.

Though rational midwifery was probably practised before the second century, it was at that time that the first work in midwifery was written. In this century Soranus of Ephesus wrote a work on midwifery and diseases of women, in which he taught sensible care and assistance to women in childbirth, based on practical and anatomical knowledge. He advocated podalic version as a means of delivering the child instead of destroying it with instruments. He however was not the first to practise this manœuvre, as it was described and practised by the Egyptians as early as 300 B.C.

PLATE I

An obstetrical chair of the fifteenth century.

Ancient methods of hastening labour.

PLATE II

An Indian brave hastening labour. Formerly among some of the tribes of American Indians labour was hastened by placing the woman on the prairie and having a horseman ride at her with the apparent intention of trampling her. Although the rider turned aside at the last moment the fear inspired in the woman was sometimes effective in shortening her labour.

The height of prudery. A physician operating under the sheet.

*With acknowledgments to Wm. Heinemann (Medical Books) Ltd.*

The custom for a physician to act as midwife ceased in western civilization in the fourth century, and from this date until a little over three hundred years ago midwifery passed into the hands of untrained midwives. Midwifery was not only ignored by the physician, but he was actually prohibited by custom and law from undertaking it. The result was that no advance in obstetrics took place until the sixteenth century, when the participation of the physician in midwifery came into limited favour again.

It is true that in the Middle Ages Roslin of Worms wrote the famous book entitled *The Garden of Roses for Pregnant Women and Midwives*. However, this is of historical interest only, in that it was the first book on midwifery written in Europe for fourteen centuries. It brought out nothing new, as one would expect, as Roslin had never seen a child born.

Modern midwifery commenced with Ambrose Paré in 1573. He re-introduced podalic version for delivery, and opposed Cæsarean section. Cæsarean section had been used for many centuries to deliver the child from the mother who died in childbirth, but there is no record of this operation having been performed on the living until the fifteenth or sixteenth centuries. In Paré's day, it was attended with a high mortality, and hence his preference for podalic version. Paré also started a school for midwives in Paris, and this was the first training-school for midwives. It was not until the eighteenth century, two hundred years later, that a similar school was started by Smellie in London.

In the seventeenth century two happenings brought the physician back into midwifery. In France the physician Boucher was summoned to attend Louis XIV's mistress, La Vallière. This apparently was a successful delivery, as in 1670 Clement, another physician, attended Madame Montespan, and in 1682 the Queen of France. These births must have been successful also, for the title of accoucheur was conferred on Clement, instead of the derisive title of "man-midwife" or "mid-man". The title of accoucheur was given to obstetricians in this country until quite modern times.

In England, at about the same period as Paré, Chamberlain invented the obstetric forceps. As these were a family secret it was necessary to call in a Chamberlain to use them, and by this means in this country also the physician came back to obstetrics. The history of the Chamberlain forceps is interesting. They were probably invented by Peter Chamberlain in 1600, and his original forceps were found in a secret cupboard in his house one hundred and fifty years after his death. Peter Chamberlain was followed by his son Hugh, who translated Mariceau's *Midwifery* into English, and visited Mariceau in Paris. While in Paris he demonstrated his forceps on one of Mariceau's patients, and unfortunately, as so often happens when one is demonstrating an operation or technique, things went wrong and the patient died from a ruptured uterus. In later years Hugh Chamberlain got mixed up in politics and the Land Bank, and in consequence lost a fortune and fled to Holland. In Holland he craftily sold one blade only of his forceps to the Dutch physician Roonhuysen : he had previously tried unsuccessfully to sell them to Mariceau. All Hugh's sons practised midwifery in England, and as the forceps were a family secret they made large fortunes.

The re-introduction of the physician into midwifery met with considerable opposition from the midwives, and Smellie was described by one as " a great horse godmother of a he-midwife ". Difficulties were also encountered from the prudery of patients, other than those in royal circles among whom modesty was not carried to this extent. Louis XIV is said to have shown his interest in the delivery of his mistress by watching the procedure from the concealment offered by some curtains. On the other hand, the early physicians were forced frequently to serve their prudish patients " under the sheet ". One end of a sheet was tied to the patient's waist, and the other around the physician's neck. He made his examinations and delivery blindly beneath this covering.

In 1651 William Harvey wrote the first scientific description of generation and labour, and in the eighteenth century Smellie published the first exact measurements of the pelvis, and introduced the modern forceps blade. In the same century induction of premature labour was introduced for contracted pelvis, though the old Hippocratic belief that a child born at seven months had a better chance of survival than one born later was still held. This belief is even now a popular superstition.

In 1858 Semmelweis in Vienna discovered that puerperal fever was caused by infection, and about the same time Oliver Wendell Holmes in America was preaching the same thing. In the same decade the work of Lister completed that which Semmelweis had started.

The introduction of antiseptics and anæsthetics began the modern advance in obstetrics, but even thirty-five years ago the examiners' table was full of instruments for the destruction of the child rather than for its delivery alive. Obstructed labour and obstetrical tragedies were not uncommon in my early days on the staff, high forceps delivery was common, and for minor degrees of flat pelvis podalic version was performed. Cæsarean section held a moderate mortality from abdominal sepsis in those days, and hence vaginal delivery, however difficult, was preferred to abdominal. At the present time obstructed labour and serious obstetrical complications are uncommon. This I feel certain is due to the following : antenatal supervision, the correct measurement of the pelvis by X-rays, the estimation of cephalo-pelvic disproportion, induction of labour and trial labour, and last but not least the modern method of delivery of breech presentation. The introduction of the Sulphonamide group of drugs and Penicillin, with improved obstetric technique, has lowered maternal and fœtal mortality to its present rate.

Turning to gynæcology, I consider that the greatest progress made in the last fifty years has been in the treatment of prolapse. In the early days of medicine it was thought that the uterus was an animal which consumed the sperm, and this was firmly believed by the fathers of medicine. This being the case it was presumed that in cases of prolapse the animal was coming out, and the treatment was to repel it by offending its sense of smell. For this purpose foul-smelling fumigations were employed. If this did not succeed, a type of pessary treatment was employed. Hippocrates advocated the insertion of half a pomegranate. Variations on this were a peeled pomegranate soaked in vinegar, a ball of wool, sponges or animal bladders.

Hippocrates also advocated a more radical treatment : this was to scarify the tissues around the uterus, and then wash the scarred tissue with linen. It is presumed that this caused infection of the tissues around the uterus with subsequent fibrosis, thus pulling up the uterus. The counterpart of this operation was advocated not many years ago, namely the injection of a sclerosing substance into the parametria, and the resulting fibrosis drew up and fixed the uterus.

However, in later years when the anatomy of the uterus was more understood pessaries came into use. In 1472 Mathieu de Garde recommended a metal pessary in the shape of a penis, to be used with astringent lotions. Paré in 1564 described three types of pessary made of various metals, but it is most probable that pessaries were in common use at a much earlier date. There is a record that in 1579 a woman was seen who had retained a cork pessary *in situ* for forty years. Also German women at this period used empty walnut-shells covered in wax, and shaped like a fowl's egg. Even in modern times strange objects are still used. I recently removed from an old lady a ping-pong ball which had been unchanged for four years. This had proved perfectly satisfactory in retaining the prolapse and was perfectly clean. I tried various types of modern pessary, all of which were unsatisfactory to the patient, and in the end I replaced the ping-pong ball, to her great delight.

The cup-and-stem pessary was invented by Jean Bauhin towards the end of the sixteenth century, and modifications of this pessary are used to this day for elderly women in whom operation is contra-indicated. Hodge introduced his pessary in the early nineteenth century and this type or modifications are still used.

Pessary treatment of prolapse was in its heyday when gynæcology first became a speciality. Some of you will remember early gynæcological out-patients—queues of women lining up waiting to have their pessaries removed, cleaned and replaced, or a new one inserted. Students came down an hour before the honorary, and this time was spent in changing "rings". What a change now! Only the occasional pessary to insert, and students even anxious to do it for the sake of practice. The reason for this is the great advance made in operations for repair of the pelvic floor. The early operations were based on suspending or fixing the uterus in the abdominal cavity, and the opening in the pelvic floor was ignored. The result was that the majority of these operations gave only temporary relief, and the prolapse recurred. More exact anatomical knowledge of the pelvic floor and the uterine supports led to the modern operations for pelvic repair. The results of these operations are probably the most gratifying to the patient and operator of any operation in gynæcology, and one gets more thanks for a successful repair than for the removal of the largest abdominal tumour. That the modern operation is successful is shown by the willingness of patients to submit to operation for this lesion, rather than avoid operation by the wearing of a pessary: the opposite was the case not so many years ago.

## Figures and Tables

**Figure f1:**
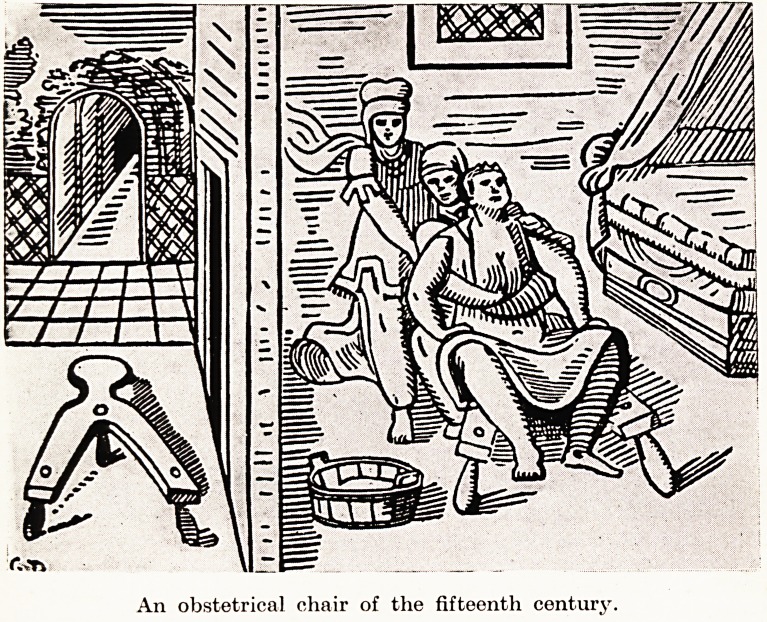


**Figure f2:**
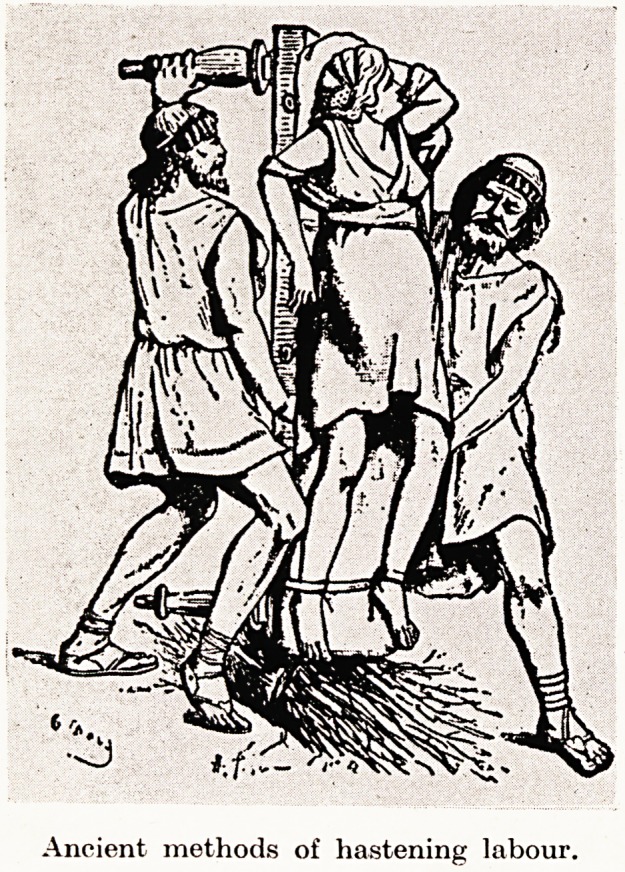


**Figure f3:**
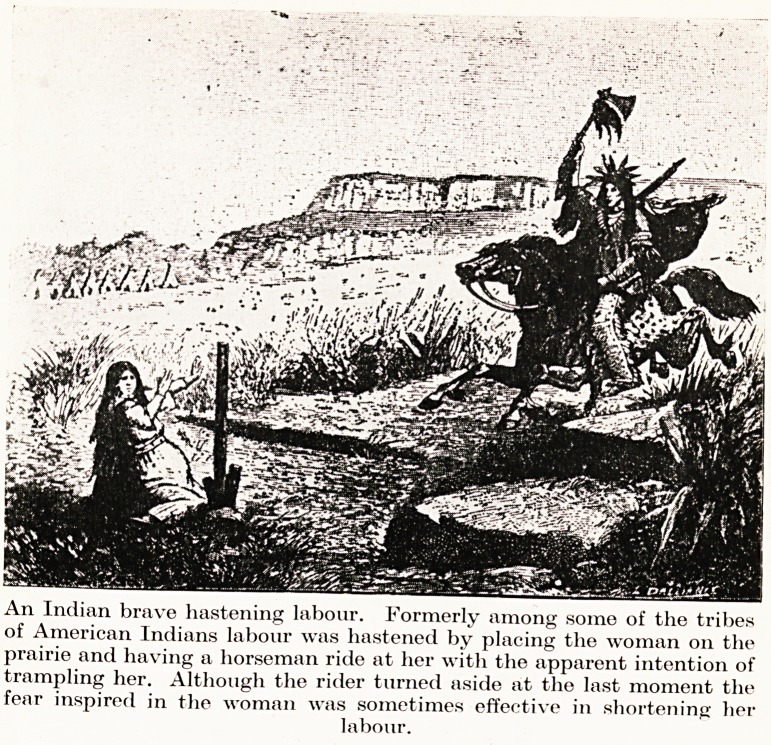


**Figure f4:**